# Psychosocial Determinants, Parental Mental Health, and Bonding in Extreme Prematurity: Results from the PremTEA Study

**DOI:** 10.1192/j.eurpsy.2026.10186

**Published:** 2026-07-31

**Authors:** A. Romero Teruel, M. J. Molina Aguilera, E. Rodríguez-Toscano, B. Almansa, S. Zeballos-Sarrato, D. Blanco-Bravo, A. Garcia-Blanco, L. Pina-Camacho, J. Merchan-Naranjo

**Affiliations:** 1 Dr. Rodríguez Lafora Hospital; 2 Gregorio Marañón General University Hospital; 3 Department of Experimental Psychology, Cognitive Processes and Speech Therapy, Complutense University of Madrid (UCM), Madrid; 4 La Fe University Hospital; 5 La Fe Health Research Institute, Valencia; 6 Neonatology Department, Gregorio Marañón General University Hospital; 7 IiSGM; 8 CIBERSAM, Madrid, Spain

## Abstract

**Introduction:**

Extreme prematurity (<28 weeks) exposes parents to high stress levels in the Neonatal Intensive Care Unit (NICU), increasing risks of anxiety, depression, and impaired bonding. The interplay between family structure, cultural background, and parental mental health trajectories remains insufficiently studied.

**Objectives:**

To examine longitudinal changes in anxiety, depression, and bonding among parents of extremely preterm infants (EPTI) versus term infants (TI), assessing the impact of family size, race/ethnicity, and social support on parental mental health outcomes.

**Methods:**

We conducted a prospective observational study including 150 EPTI and 50 TI. Parental anxiety (State-Trait Anxiety Inventory, STAI), depression (Beck Depression Inventory-II, BDI-II), NICU stress (PSS:NICU), bonding (Postpartum Bonding Questionnaire), and perceived social support were assessed at birth and at 40 postmenstrual weeks (PMW). Analyses included Kruskal–Wallis tests, Spearman correlations, hierarchical clustering, and mixed-effects regression models.

**Results:**

Anxiety and depression scores decreased significantly from birth to 40 PMW across most family structures (STAI: β = −19 to −25, p < 0.001; BDI-II: β = −3.5 to −8.1, p ≤ 0.043). Families with 2–3 cohabitants showed the greatest symptom reduction, while larger households (>5) exhibited persistently higher scores. Non-Caucasian ethnicity and lower maternal education were associated with elevated distress and weaker bonding. Heatmaps and clustering revealed distinct psychosocial profiles linked to family size and cultural background. Longitudinal models confirmed time as a significant predictor of symptom reduction in most subgroups.

**Conclusions:**

Parental mental health trajectories after extreme prematurity are shaped by family structure, cultural background, and social support. Culturally sensitive, family-focused interventions in NICUs may mitigate long-term psychological burden and improve bonding outcomes.

**Disclosure of Interest:**

None Declared

